# Plasma amino acid metabolic alterations and their associations with ocular parameters in early primary angle-closure glaucoma

**DOI:** 10.3389/fmed.2026.1928552

**Published:** 2026-09-15

**Authors:** Hui Liu, Xiaoli Liu, Shuang Liang, Yuxin Geng, Hui Bu, Danyan Liu

**Affiliations:** 1 Hebei Medical University Clinical Medicine Postdoctoral Research Station (The Second Hospital), Shijiazhuang, Hebei, China; 2 Department of Ophthalmology, The Second Hospital of Hebei Medical University, Shijiazhuang, Hebei, China; 3 Department of Neurology, The Second Hospital of Hebei Medical University, Shijiazhuang, Hebei, China

**Keywords:** amino acids, metabolomics, plasma, primary angle-closure glaucoma, swept source optical coherence tomography angiography

## Abstract

**Purpose:**

To explore the plasma metabolite profiles of patients with early primary angle-closure glaucoma (PACG) and investigate their relevance to ocular characteristics and retinal-associated parameters.

**Methods:**

Plasma samples were obtained from 30 early PACG patients and 30 healthy controls for untargeted and targeted metabolomics analysis. Additionally, relevant clinical data such as ocular characteristics and retinal-associated parameters were collected. Correlations between clinical data and differential metabolites were further analyzed.

**Results:**

A total of 439 differential metabolites were identified through untargeted metabolomics analysis, with amino acids being the most abundant category among the differential metabolites. Amino acid-targeted metabolomics revealed that L-alanine, L-lysine, citrulline, L-tyrosine, and glycylglycine were significantly elevated in early PACG (all *P* < 0.05). In addition, intraocular pressure (IOP) was significantly higher (20.9 ± 2.5 vs. 14.2 ± 2.4 mmHg, *P* < 0.001), axial length (AL) was shorter (22.8 ± 0.1 vs. 23.2 ± 0.2 mm, *P* < 0.001), and lens thickness (LT) was greater (4.8 ± 0.2 vs. 4.3 ± 0.2 mm, *P* < 0.001) in early PACG patients than in healthy controls. Reduced retinal nerve fiber layer (RNFL) thickness, ganglion cell complex (GCC) thickness, and superficial vascular complex vessel density in the peripapillary and macular regions were observed in early PACG patients compared with healthy controls (all *P* < 0.05). We further identified five amino acids that showed a positive correlation with IOP and LT, and a negative correlation with AL, as well as with the thickness of the RNFL and GCC in the central 3–6 mm annular region (all *P* < 0.05).

**Conclusion:**

The plasma of early PACG patients showed distinctive metabolic profiles, which provided new insights into the pathogenesis of the early phase of PACG patients.

## 1 Introduction

Glaucoma is a chronic optic neuropathy characterized by progressive loss of retinal ganglion cells and irreversible visual field deficiency (1). Primary angle-closure glaucoma (PACG) is the predominant form of glaucoma in Asia. With the continuous advancement and increasing clinical availability of optical coherence tomography (OCT) and OCT angiography (OCTA), a large number of studies have been performed on retinal nerve fiber layer (RNFL) thickness and vessel density around the optic nerve head in glaucoma patients (2–5). Additionally, retinal vascular changes in the macula of PACG patients have been analyzed by using OCTA (2). However, in view of the complex pathogenesis of PACG and the risk of severe visual impairment associated with the disease, focusing solely on ocular manifestations, such as RNFL thinning and reduced retinal blood flow, may be insufficient for comprehensively understanding the underlying mechanisms of PACG. Consequently, it is necessary to explore specific potential biomarkers associated with PACG.

Proteomics and transcriptomics have been widely applied to investigate the pathogenesis of glaucoma using human peripheral blood, retinal cell models, and animal retinal ganglion cells (6–8). Although plasma metabolomic studies have been conducted in PACG, metabolic alterations specifically associated with early-stage disease remain insufficiently characterized. Metabolomics has unique advantages. On the one hand, metabolites are easily affected by external factors, and their changes are more sensitive than genes or proteins (9, 10). On the other hand, metabolomics is most strongly linked to disease-related phenotypes (11). However, the metabolome is highly dynamic and can also be influenced by factors such as stress, comorbidities, and lifestyle. These advantages and limitations collectively indicate that plasma metabolomics can provide new insights into a better understanding of the underlying pathophysiological mechanisms of early PACG.

In this study, we used untargeted and targeted metabolomics analysis to characterize the plasma metabolic profiles of patients with early PACG. Furthermore, swept source OCTA (SS-OCTA) was utilized to evaluate the retinal vascular density and ganglion cell complex (GCC) thickness in the peripapillary and 1–12 mm annular area of macula among patients with early PACG. The main aim of the study was to characterize the plasma metabolite profiles of early PACG to comprehensively understand the pathological alterations in PACG. Additionally, we further analyzed the correlations between differential metabolites with ocular characteristics and retinal-associated parameters.

## 2 Materials and methods

### 2.1 Subjects

A total of 60 participants, including 30 patients with early PACG (30 eyes) and 30 healthy controls (30 eyes), were recruited from the Second Hospital of Hebei Medical University between March 2025 and September 2025. PACG was defined as more than 180° of iridotrabecular contact, elevated intraocular pressure (IOP) or peripheral anterior synechiae, and with glaucomatous optic neuropathy (12, 13). According to the Hodapp-Anderson-Parrish 2 criteria (14), patients with early PACG were recruited in this study. Exclusion criteria included trauma, history of intraocular surgery and YAG laser peripheral iridotomy prior to completing the relevant examinations of this study, equivalent refractive error > 6.0 diopters (D), secondary angle closure caused by other factors, and any other ocular disorder. All patients with early PACG were treated with topical antiglaucoma agents to control IOP. All participants signed the informed consent before recruitment. The study complied with the Declaration of Helsinki and was approved by the Institutional Review Board of the Second Hospital of Hebei Medical University.

### 2.2 Clinical parameter measurement

All participants underwent comprehensive ophthalmic examinations, including IOP, slit-lamp examinations, fundus examinations, gonioscopy, axial length (AL), anterior chamber depth (ACD), and lens thickness (LT). Patients with early PACG underwent visual field examination using the 24-2 Swedish Interactive Threshold Algorithm (SITA) standard strategy (Carl Zeiss, Germany).

All participants underwent OCTA examinations (VG200D; DVision Imaging, Henan, China) performed by experienced ophthalmologists. Each participant underwent an optic disc scan covering an area of 6 × 6 mm and a macula scan covering an area of 12 × 12 mm (Figure 1). The retinal-associated parameters were measured as an annular ring with a diameter of 2–4 mm around the peripapillary region. Based on the research of Garway-Heath et al. (15), the annular ring was automatically divided into eight anatomical locations by built-in software, including the nasal superior (NS), nasal inferior (NI), inferior nasal (IN), inferior temporal (IT), temporal inferior (TI), temporal superior (TS), superior temporal (ST), and superior nasal (SN) areas. The macular scan was divided into annular regions with diameters of 1–3 mm, 3–6 mm, 6–9 mm, and 9–12 mm in accordance with the Early Treatment Diabetic Retinopathy Study (ETDRS), and each circumferential annular area was further divided into four quadrants: superior, inferior, temporal, and nasal quadrants. The built-in software of the OCTA device automatically calculated the mean thickness of the RNFL, ganglion cell-inner plexiform layer (GC-IPL), and GCC among the macular and peripapillary regions. Moreover, the OCTA scan generates and segments the retina into four layers of vascular plexuses containing the radial peripapillary capillary plexus (RPCP), the superficial vascular plexus (SVP), the intermediate capillary plexus (ICP), and the deep capillary plexus (DCP). As these four layers of vascular plexuses are very thin and difficult to distinguish in some anatomical locations, we combined the RPCP and SVP into the superficial vascular complex (SVC) and the ICP and DCP into the deep vascular complex (DVC). In addition, all participants underwent the 3 × 3 mm scan of macula to obtain foveal avascular zone (FAZ) size. In view of the interference of large vessels around the peripapillary region, retinal-associated parameters from nasal quadrant with the diameter beyond 6 mm were excluded. Only one eligible eye from each participant was included in the analysis.

**Figure 1:**
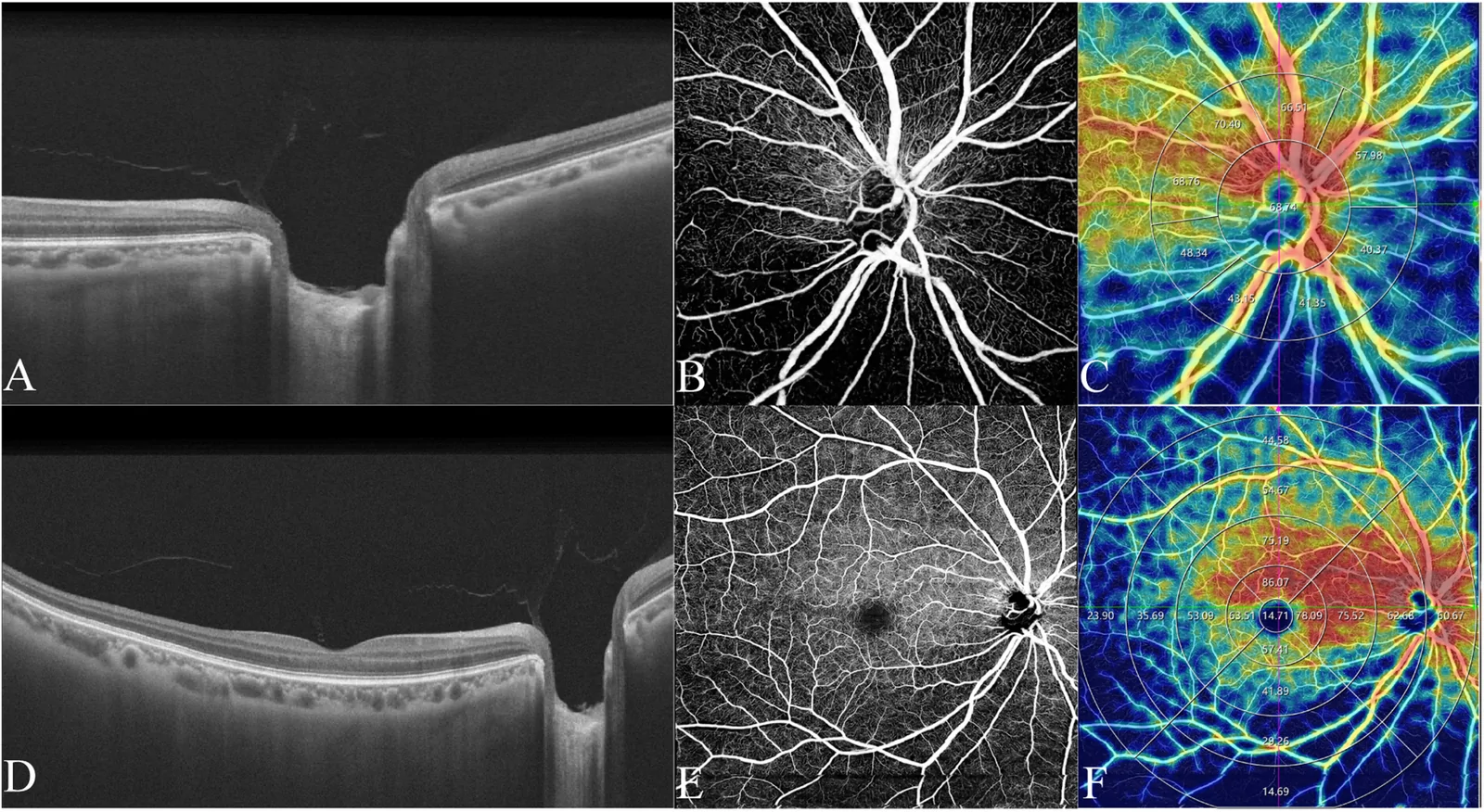
Representative case of the optic disc scan and macula scan of an eye with early PACG. The 6 × 6 mm scan of the optic disc area in the early PACG eye **(A–C)** showed B-scan image **(A)**, SS-OCTA image **(B)**, and the annular area was divided into eight anatomical locations according to the study of Garway-Heath **(C)** The 12 × 12 mm scan of the macular area in the early PACG eye **(D–F)** showed B-scan image **(D)**, SS-OCTA image **(E)**, and anatomical divisions according to ETDRS **(F)** PACG, primary angle-closure glaucoma; SS-OCTA, swept source optical coherence tomography angiography; ETDRS, Early Treatment Diabetic Retinopathy Study.

### 2.3 Sample collection and metabolomics analysis

Blood samples were collected in the morning after overnight fasting before any food intake and placed into heparin tubes. The tubes were immediately placed on ice and centrifuged at 2,000 g for 10 min at 4 °C to collect plasma and stored at −80 °C until metabolites profiling.

Plasma untargeted metabolomics analysis was performed by Novogene Co. (Beijing, China). After all plasma samples were thawed at 4 °C, 100 *μ*L plasma was mixed with 400 *μ*L of 80% methanol, which was then incubated on ice for 5 min and centrifuged at 15,000 g, 4 °C for 20 min. A portion of the supernatant was diluted with LC-MS grade water to a final concentration containing 53% methanol, then transferred to a new Eppendorf tube and centrifuged at 15,000 g for 20 min at 4 °C. The final supernatant was injected into the LC-MS/MS system for analysis (16, 17). UHPLC-MS/MS analyses were performed using a Vanquish UHPLC system (Thermo Fisher, Germany) coupled with an Orbitrap Q Exactive™ HF mass spectrometer (Thermo Fisher, Germany) according to previously described procedures (18). Chromatographic separation was achieved using a Hypersil Gold column (100 × 2.1 mm, 1.9 *μ*m) at a flow rate of 0.2 mL/min. The mobile phase system consisted of solvent A (0.1% formic acid in water) and solvent B (methanol). The elution gradient was programmed as follows: solvent B was held at 2% for 1.5 min, increased from 2% to 85% between 1.5 and 3 min, further increased to 100% from 3 to 10 min, returned to 2% at 10.1 min, and maintained until the completion of the 12 min run. The mass spectrometer was operated in both positive and negative ionization modes with a spray voltage of 3.5 kV, capillary temperature of 320 °C, sheath gas flow rate of 35 psi, auxiliary gas flow rate of 10 L/min, S-lens RF level of 60, and auxiliary gas heater temperature of 350 °C. The raw UHPLC-MS/MS data were preprocessed by XCMS software to perform peak alignment, peak picking, and quantitation for each metabolite. Missing values were handled according to the standard preprocessing procedures of the metabolomics workflow before statistical analysis. The identified metabolites were annotated using the Kyoto Encyclopedia of Genes and Genomes (KEGG) database (https://www.genome.jp/kegg/pathway.html), HMDB database (httpshmdb.ca/metabolites), and LIPMaps database (http://wwwipidmaps.org/). The variable important in projection (VIP) value of each metabolite was calculated by partial least squares discriminant analysis (PLS-DA). The *t*-test was used to calculate the statistical significance (*P* value) of each metabolite between early PACG patients and healthy controls, and the fold change (FC) was calculated. Multiple testing correction was performed using the Benjamini–Hochberg procedure, and metabolites with an FDR-adjusted *P* value < 0.05 were considered statistically significant. Differential metabolites were identified by combining VIP values, FDR-adjusted *P* values, and FC criteria. Metabolites simultaneously meeting the criteria of VIP > 1, FDR-adjusted *P* value < 0.05, and FC > 1.2 or <0.833 were considered significantly altered.

Within the 60 participants who underwent untargeted metabolomics profiling, plasma samples from a randomly selected subset of 20 early PACG patients and 20 healthy controls were further analyzed using amino acid-targeted quantitative analysis based on the ultra-high performance liquid chromatography coupled to tandem mass spectrometry (UHPLC-MS/MS) system (ExionLC™ AD UHPLC-QTRAP® 6500+, AB SCIEX Corp., Boston, MA, USA). The mobile phase was composed of 0.1% formic acid in 5 mM ammonium acetate as solvent A and 0.1% formic acid in acetonitrile as solvent B. The solvent gradient was set as follows: initial 90% B, 1.0 min; 90%–85% B, 2.0 min; 85%–75% B, 3.5 min; 75%–70% B, 7.0 min; 70%–45% B, 10.0 min; 45%–90% B, 11.1 min; 90% B, 13.0 min.

### 2.4 Statistical analysis

Statistical analysis was performed using SPSS version 25.0 or GraphPad Prism version 9.0. Data were presented as means ± standard deviations, medians and interquartile range, or numbers and percentages, as appropriate. Volcano plots were generated to visualize differential metabolites based on log2 (FC) and −log10 (FDR-adjusted *P* values) using the ggplot2 package in R. Pathways enrichment analysis of differential metabolites was performed using the KEGG database.

As for clinical manifestations and retinal-associated parameters, quantitative variables were analyzed using independent samples *t*-test, while categorical variables were analyzed using *χ*^2^ test. The Spearman correlation analysis was then performed on the aforementioned screened differential metabolites and clinical indicators, and a heat map was formed. *P* value less than 0.05 was considered statistically significant.

## 3 Results

### 3.1 Study participants characteristics

A total of 60 participants were enrolled in this study, including 30 patients with early PACG and 30 healthy controls. The general conditions and clinical characteristics of the two groups are shown in Table 1. There were no significant differences with regard to gender, hypertension, diabetes mellitus, body mass index and age between both groups. Generally, higher IOP, shorter AL, shallower ACD, and thicker LT were observed in patients with early PACG, as compared with healthy controls (all *P* < 0.001).

**Table 1:** Demographic and clinical characteristics of study participants.

Items	PACG (*n* = 30 eyes)	Healthy control (*n* = 30 eyes)	*P* value
Age, mean ± SD, y	57.9 ± 7.6	56.0 ± 8.9	0.362^¶^
Male gender, *n* (%)	13/30 (43.3%)	16/30 (53.3%)	0.606^†^
Hypertension, *n* (%)	2/30 (6.7%)	1/30 (3.3%)	>0.999^†^
Diabetes mellitus, *n* (%)	1/30 (3.3%)	3/30 (10.0%)	0.612^†^
Body mass index, mean ± SD, kg/m^2^	23.3 ± 2.3	23.4 ± 1.8	0.891^¶^
Intraocular pressure, mean ± SD, mmHg	20.9 ± 2.5	14.2 ± 2.4	<0.001^¶^
Axial length, mean ± SD, mm	22.8 ± 0.1	23.2 ± 0.2	<0.001^¶^
Anterior chamber depth, mean ± SD, mm	1.8 ± 0.3	2.5 ± 0.3	<0.001^¶^
Lens thickness, mean ± SD, mm	4.8 ± 0.2	4.3 ± 0.2	<0.001^¶^
Visual field mean deviation, mean ± SD, dB	−2.5 ± 1.3	—	NA

PACG, primary angle-closure glaucoma; NA, not applicable; SD, standard deviation.

¶ Statistical differences were assessed by independent samples *t*-test.

† Statistical differences were assessed by *χ*^2^ test or Fisher exact test, as appropriate.

In the 12 × 12 mm macular scan (Supplementary Table S1), the mean RNFL thickness, mean GCC thickness, and mean SVC vessel density in annular regions with diameters of 1–3 mm, 3–6 mm, and 6–9 mm, respectively, were lower in early PACG patients than in healthy controls (all *P* < 0.001). Lower mean GC-IPL thickness of the 1–3 mm and 3–6 mm annular regions was noted in early PACG patients than in healthy controls (both *P* < 0.01). There was no significant difference concerning the mean DVC vessel density of the four annular regions between both groups. Furthermore, the size of FAZ in early PACG patients was larger than that in the healthy controls (*P* < 0.001). As for the 6 × 6 mm optic disc scan (Supplementary Table S2), the mean RNFL thickness, mean GCC thickness, and mean SVC vessel density were all found to be reduced in patients with early PACG as compared with healthy controls (all *P* < 0.01). However, there were no differences regarding the mean GC-IPL thickness and mean DVC vessel density between both groups.

### 3.2 Untargeted metabolite results

A total of 2,047 metabolites were identified in the positive mode of untargeted metabolomics analysis, while a total of 1,203 metabolites were identified in the negative mode. The supervised statistical method PLS-DA was used to characterize the metabolic profiles in both positive and negative modes to evaluate metabolic differences between the two groups (Figure 2). A total of 439 significantly differential metabolites were identified based on the predefined threshold criteria, including 243 significantly upregulated and 196 significantly downregulated metabolites (Figure 3A). All differential metabolites were categorized according to the three-level classification of metabolites, with amino acids being the most abundant category among the differential metabolites. (Figure 3B). The differential metabolites between early PACG and healthy controls were enriched in pathways such as metabolic pathways, neuroactive ligand-receptor interaction, glycine, serine and threonine metabolism, as well as cysteine and methionine metabolism (Figure 3C).

**Figure 2:**
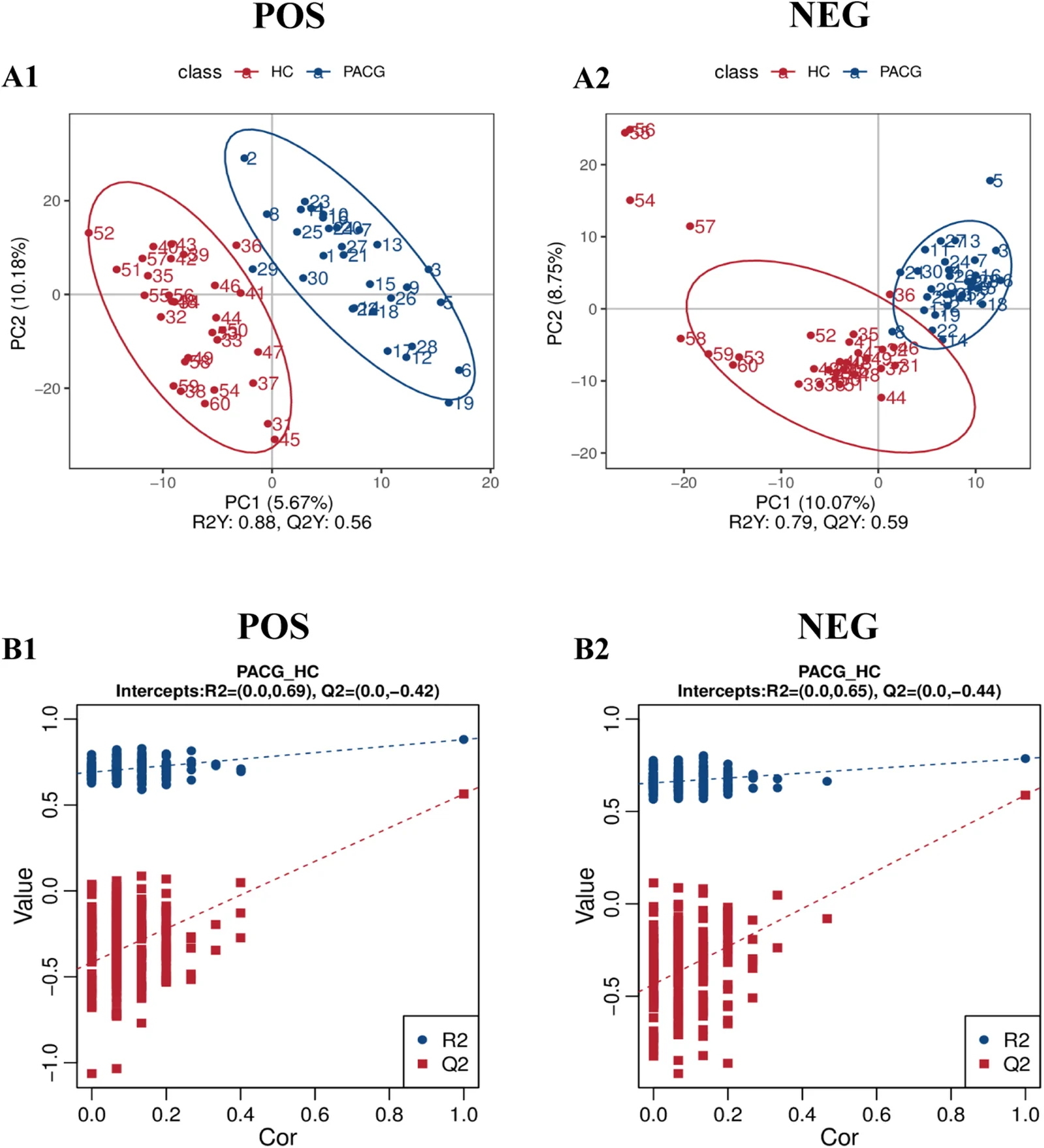
PLS-DA between the two groups. PLS-DA score **(A1)** and PLS-DA valid **(B1)** in positive mode for early PACG patients vs. HC. PLS-DA score **(A2)** and PLS-DA valid **(B2)** in negative mode for early PACG patients vs. HC. PLS-DA, partial least square discriminant analysis; PACG, primary angle-closure glaucoma; HC, healthy controls.

**Figure 3:**
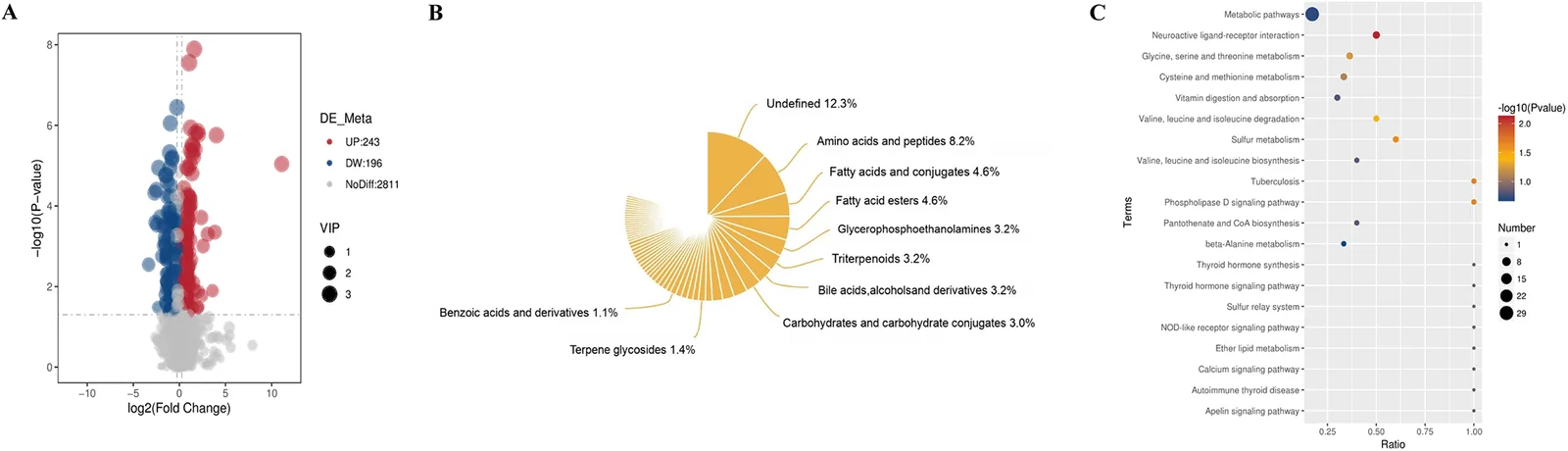
Untargeted metabolomics analysis results. Volcano plot **(A)** of differential metabolites between both groups. Pie chart **(B)** of all differential metabolites classified by metabolite Class III. KEGG pathway enrichment **(C)** of differential metabolites.

### 3.3 Targeted metabolite results

A randomly selected subset of plasma samples from 20 early PACG patients and 20 healthy controls within the untargeted metabolomics cohort was further analyzed using amino acid-targeted metabolomics, with demographic and clinical characteristics summarized in Supplementary Table S3. This analysis was performed to characterize amino acid metabolic alterations associated with early PACG. The levels of L-alanine, L-lysine, citrulline, L-tyrosine, and glycylglycine were significantly higher in patients with early PACG as compared with the healthy controls (all *P* < 0.05) (Figure 4). However, the results showed that the amino acids enriched in the aforementioned pathways were not detected in the plasma samples.

**Figure 4:**
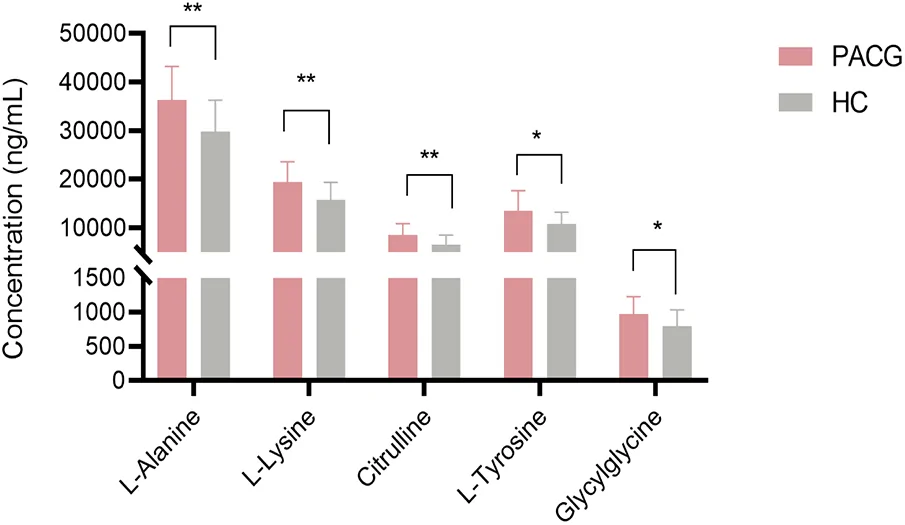
Plasma levels of L-alanine, L-lysine, citrulline, L-tyrosine, and glycylglycine between the two groups. *P < 0.05; **P < 0.01. PACG, primary angle-closure glaucoma; HC, healthy controls.

### 3.4 Correlation analysis between five amino acids and clinical data

Based on amino acid-targeted metabolomics analysis, 5 amino acids (L-alanine, L-lysine, citrulline, L-tyrosine, and glycylglycine) were screened for correlation analysis with clinical data (Figure 5). All five amino acids were positively correlated with IOP and LT, while negatively correlated with AL and the RNFL thickness and GCC thickness in the 3–6 mm annular region (all *P* < 0.05).

**Figure 5:**
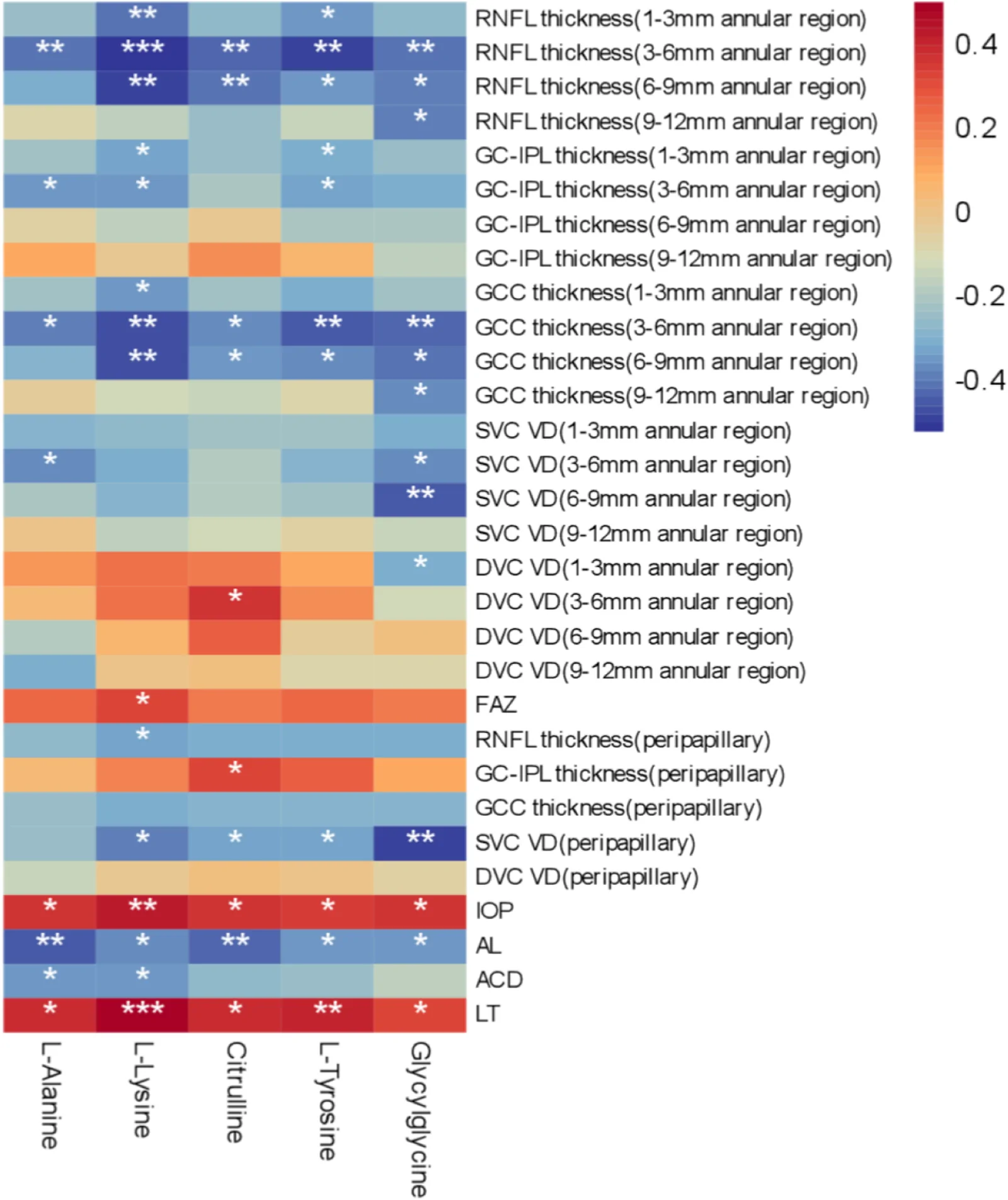
Heatmap of correlation analysis between retinal-associated parameters and ocular characteristics with differential metabolites. The X-axis represents the differential metabolites, and the Y-axis represents the retinal-associated parameters and ocular characteristics. Red indicates positive correlation, while blue indicates negative correlation. The darker the color, the stronger the correlation. *P < 0.05; **P < 0.01; ***P < 0.001. RNFL, retinal nerve fiber layer; GC-IPL, ganglion cell-inner plexiform layer; GCC, ganglion cell complex; SVC, superficial vascular complex; DVC, deep vascular complex; FAZ, foveal avascular zone; IOP, intraocular pressure; AL, axial length; ACD, anterior chamber depth; LT, lens thickness.

## 4 Discussion

In this study, both untargeted and targeted metabolomics approaches were applied to characterize the plasma metabolite profile of early PACG patients. The plasma levels of L-alanine, L-lysine, citrulline, L-tyrosine, and glycylglycine in early PACG patients were significantly increased compared with those in healthy controls. Meanwhile, we used SS-OCTA to evaluate the changes in retinal-associated parameters of the macula and peripapillary regions among early PACG patients. Compared with the healthy controls, the mean thickness of RNFL and GCC as well as the mean SCV vessel density in the 1–3 mm, 3–6 mm, and 6–9 mm annular regions of the macula and the peripapillary region were decreased in early PACG patients. Furthermore, the present study innovatively assessed the correlation between plasma metabolites and clinical data in early PACG patients. All five amino acids were positively correlated with IOP and LT, and negatively correlated with AL as well as the mean thickness of RNFL and GCC in the 3–6 mm annular region.

Glaucoma, as the leading cause of irreversible blindness worldwide, is a major public health problem. With the continuous improvement in the understanding of the pathological mechanisms underlying glaucoma, coupled with the development and innovation of examination equipment, the level of diagnosis and treatment for glaucoma has been remarkably enhanced. Aqueous humor analysis enables a more accurate reflection of intraocular biochemical changes. However, it is difficult to obtain aqueous humor samples from certain early PACG patients as they do not require surgical intervention, which limits the evaluation of the biochemical process of glaucoma in the early phase. Plasma, as an easily accessible biological sample, offers a novel perspective and crucial evidence for research on early-phase glaucoma through its metabolomic characteristics. In recent years, metabolomics has been widely utilized in glaucoma (19–22). Previous studies have mainly focused on either untargeted or targeted metabolomics approaches in glaucoma (23, 24). In the present study, we performed untargeted metabolomics followed by targeted amino acid quantification to characterize plasma metabolic alterations in early PACG. Untargeted metabolomics identified 439 differential metabolites in plasma samples from 60 participants, with amino acids representing the largest proportion among these metabolites. Subsequently, amino acid-targeted metabolomics was performed in a subset of 40 participants, revealing increased plasma levels of five amino acids in early PACG patients compared with healthy controls. These results demonstrated that PACG is associated with disturbances in amino acid metabolism, and this finding is consistent with a previous study (22).

L-alanine is one of the crucial amino acids that make up human proteins. Research has shown that alanine levels are significantly increased in the aqueous humor of primary open-angle glaucoma patients (25). Consistent with this finding, our study also found raised L-alanine levels in the plasma of early PACG patients. Given that metabolic alterations and mitochondrial dysfunction have been implicated in glaucomatous neurodegeneration, altered alanine metabolism may reflect metabolic disturbances associated with glaucoma pathogenesis (26, 27).

L-lysine, as an essential amino acid for the human body, is an important basic element for maintaining life activities. We found that L-lysine levels were raised in patients with early PACG, which is in agreement with a previous report (28). Lysine plays an important role in extracellular matrix (ECM) organization through its involvement in collagen synthesis and cross-linking. Given that ECM remodeling in the trabecular meshwork is closely associated with aqueous humor outflow resistance and IOP elevation, altered lysine metabolism may contribute to ECM-related metabolic changes in PACG (29).

Citrulline is an intermediate of the arginine–nitric oxide (NO) pathway and is involved in NO synthesis. Our study revealed significantly increased plasma citrulline levels in early PACG patients compared with healthy controls, suggesting altered arginine metabolism in early PACG. Given that NO is involved in vascular regulation and ocular perfusion, alterations in citrulline metabolism may affect NO-related vascular homeostasis and be associated with glaucomatous optic nerve injury (30). Further studies are needed to clarify the role of citrulline metabolism in PACG.

L-tyrosine, a precursor of catecholamines involved in neuronal function and oxidative stress regulation, was found to be increased in the plasma of early PACG patients in our study. This finding is consistent with previous studies reporting elevated plasma tyrosine levels in patients with primary open-angle glaucoma and exfoliation syndrome (31–33). Given the involvement of oxidative stress and neuronal metabolic dysfunction in glaucomatous neurodegeneration, altered tyrosine metabolism may reflect metabolic disturbances associated with glaucoma. Further studies are warranted to determine the biological significance of tyrosine alterations in PACG.

Glycylglycine is a dipeptide composed of two glycine molecules and is involved in glycine-related metabolism. Our study identified significantly elevated plasma glycylglycine levels in early PACG patients. Given that metabolic dysregulation is increasingly recognized as an important component of glaucomatous neurodegeneration, altered glycylglycine levels may reflect disturbances in small peptide metabolism associated with early PACG. However, the biological significance of glycylglycine alterations in PACG remains unclear and requires further investigation. Several studies have shown that RNFL thickness and vessel density of the central 1–6 mm circumferential annular area and peripapillary region were decreased in PACG patients, which is consistent with our results (2, 34, 35). Our results further revealed that lower thickness of RNFL and GCC, and decreased SVC vessel density were noted in the peripheral 6–9 mm annular region of early PACG patients as compared with healthy controls. More importantly, we calculated correlations between retinal-associated parameters and five amino acids and found that the thickness of RNFL and GCC in the 3–6 mm and 6–9 mm annular regions had negative correlations with L-lysine, citrulline, L-tyrosine, and glycylglycine. Additionally, we also confirmed that all five amino acids were positively correlated with IOP and LT, while negatively correlated with AL. Although we reported that these metabolites were significantly correlated with clinical characteristics, their specific roles in early PACG remain uncertain. Therefore, future studies may provide new theoretical bases.

The study has some limitations. First, the number of early PACG patients enrolled was insufficient, and owing to the limited volume of plasma samples, we only performed amino acid-targeted metabolomics analysis on partially enrolled early PACG patients. Second, the patients enrolled in this study were all Chinese from the same ophthalmology center, and the results need to be validated in other populations to broaden our understanding.

## 5 Conclusion

In conclusion, we characterized the plasma metabolite profiles of early PACG patients and identified that L-alanine, L-lysine, citrulline, L-tyrosine, and glycylglycine may play crucial roles in the disease. Furthermore, through SS-OCTA to evaluate retinal-associated parameters in early PACG patients, we found that the aforementioned metabolites were associated with RNFL and GCC thickness in the central (3–6 mm) annular region, as well as with IOP, AL, and LT.

## Data Availability

The datasets presented in this study can be found in online repositories. The names of the repository/repositories and accession number(s) can be found below: https://www.ebi.ac.uk/metabolights/, MTBLS13608.
