# Supplementary material for: Plasma amino acid metabolic alterations and their associations with ocular parameters in early primary angle-closure glaucoma
Source: Front Med (Lausanne). 2026 Sep 15;13:1928552. doi: 10.3389/fmed.2026.1928552 (PMC13619409; doi:10.3389/fmed.2026.1928552)
Supplement: Supplementary file 1 [file Supplementaryfile1.docx]

**Table S1. Comparison of Macular Optical Coherence Tomography Angiography Characteristics between PACG and Healthy Controls**

| Items | Regions | Quadrants | PACG  (n=30 eyes) | Healthy control  (n=30 eyes) | *P* Value* |
| --- | --- | --- | --- | --- | --- |
| RNFL thickness,  mean ± SD, μm | 1-3 mm | Superior | 24.6 ± 6.7 | 32.4 ± 6.3 | <0.001 |
|  |  | Temporal | 19.1 ± 3.3 | 22.2 ± 3.2 | <0.001 |
|  |  | Inferior | 24.2 ± 5.5 | 31.5 ± 5.4 | <0.001 |
|  |  | Nasal | 23.0 ± 5.0 | 28.8 ± 4.2 | <0.001 |
|  |  | Mean | 22.7 ± 4.3 | 28.7 ± 3.4 | <0.001 |
|  | 3-6 mm | Superior | 35.4 ± 10.8 | 49.3 ± 5.9 | <0.001 |
|  |  | Temporal | 20.2 ± 3.6 | 36.1 ± 12.1 | <0.001 |
|  |  | Inferior | 34.5 ± 13.4 | 50.1 ± 7.0 | <0.001 |
|  |  | Nasal | 43.1 ± 12.9 | 58.8 ± 6.3 | <0.001 |
|  |  | Mean | 33.3 ± 9.7 | 48.6 ± 4.1 | <0.001 |
|  | 6-9 mm | Superior | 40.2 ± 15.4 | 60.3 ± 13.1 | <0.001 |
|  |  | Temporal | 21.3 ± 4.1 | 32.8 ± 7.7 | <0.001 |
|  |  | Inferior | 44.9 ± 22.3 | 63.8 ± 12.9 | <0.001 |
|  |  | Mean | 35.5 ± 13.4 | 52.3 ± 8.0 | <0.001 |
|  | 9-12 mm | Superior | 42.7 ± 17.0 | 44.6 ± 16.8 | 0.668 |
|  |  | Temporal | 22.5 ± 4.6 | 27.4 ± 5.3 | <0.001 |
|  |  | Inferior | 34.0 ± 14.8 | 40.3 ± 14.0 | 0.095 |
|  |  | Mean | 33.1 ± 11.7 | 37.4 ± 11.6 | 0.151 |
| GC-IPL thickness,  mean ± SD, μm | 1-3 mm | Superior | 69.2 ± 16.8 | 82.9 ± 9.2 | <0.001 |
|  |  | Temporal | 61.8 ± 19.1 | 73.8 ± 11.2 | 0.004 |
|  |  | Inferior | 66.4 ± 18.9 | 78.2 ± 11.9 | 0.005 |
|  |  | Nasal | 68.2 ± 16.7 | 80.4 ± 8.0 | 0.001 |
|  |  | Mean | 66.4 ± 16.8 | 78.8 ± 8.3 | 0.001 |
|  | 3-6 mm | Superior | 49.4 ± 8.9 | 58.8 ± 9.3 | <0.001 |
|  |  | Temporal | 50.2 ± 13.3 | 63.9 ± 10.6 | <0.001 |
|  |  | Inferior | 47.4 ± 11.6 | 55.2 ± 8.2 | 0.004 |
|  |  | Nasal | 57.2 ± 8.1 | 66.5 ± 10.0 | <0.001 |
|  |  | Mean | 51.0 ± 9.1 | 61.1 ± 8.8 | <0.001 |
|  | 6-9 mm | Superior | 32.2 ± 4.1 | 31.5 ± 3.5 | 0.472 |
|  |  | Temporal | 32.3 ± 6.2 | 32.8 ± 5.5 | 0.734 |
|  |  | Inferior | 29.8 ± 4.6 | 28.0 ± 3.1 | 0.079 |
|  |  | Mean | 31.5 ± 3.8 | 30.8 ± 3.6 | 0.489 |
|  | 9-12 mm | Superior | 27.3 ± 2.0 | 26.9 ± 2.7 | 0.579 |
|  |  | Temporal | 24.9 ± 3.7 | 25.8 ± 3.3 | 0.313 |
|  |  | Inferior | 28.3 ± 22.2 | 23.9 ± 2.9 | 0.278 |
|  |  | Mean | 26.8 ± 7.8 | 25.5 ± 2.4 | 0.388 |
| GCC thickness,  mean ± SD, μm | 1-3 mm | Superior | 93.8 ± 22.3 | 115.3 ± 12.5 | <0.001 |
|  |  | Temporal | 80.9 ± 21.2 | 96.0 ± 11.1 | 0.001 |
|  |  | Inferior | 90.7 ± 23.8 | 109.7 ± 14.9 | 0.001 |
|  |  | Nasal | 91.2 ± 20.5 | 109.2 ± 10.0 | <0.001 |
|  |  | Mean | 89.2 ± 20.2 | 107.5 ± 8.9 | <0.001 |
|  | 3-6 mm | Superior | 84.8 ± 19.1 | 108.1 ± 14.0 | <0.001 |
|  |  | Temporal | 70.4 ± 15.8 | 100.0 ± 10.8 | <0.001 |
|  |  | Inferior | 81.8 ± 20.0 | 105.3 ± 13.1 | <0.001 |
|  |  | Nasal | 100.3 ± 20.2 | 125.2 ± 14.5 | <0.001 |
|  |  | Mean | 84.3 ± 17.7 | 109.7 ± 10.6 | <0.001 |
|  | 6-9 mm | Superior | 72.4 ± 17.5 | 91.8 ± 15.0 | <0.001 |
|  |  | Temporal | 53.6 ± 8.7 | 65.6 ± 8.1 | <0.001 |
|  |  | Inferior | 74.8 ± 22.1 | 91.8 ± 14.7 | 0.001 |
|  |  | Mean | 66.9 ± 15.4 | 83.1 ± 10.2 | <0.001 |
|  | 9-12 mm | Superior | 70.0 ± 17.3 | 71.5 ± 16.9 | 0.728 |
|  |  | Temporal | 47.4 ± 6.8 | 53.2 ± 7.3 | 0.002 |
|  |  | Inferior | 62.3 ± 20.7 | 64.2 ± 15.1 | 0.696 |
|  |  | Mean | 59.9 ± 12.2 | 63.0 ± 12.4 | 0.335 |
| SVC vessel density,  mean ± SD, % | 1-3 mm | Superior | 29.1 ± 9.2 | 44.9 ± 7.8 | <0.001 |
|  |  | Temporal | 21.7 ± 7.3 | 34.1 ± 5.7 | <0.001 |
|  |  | Inferior | 25.5 ± 11.0 | 41.0 ± 8.5 | <0.001 |
|  |  | Nasal | 26.3 ± 8.2 | 37.8 ± 8.4 | <0.001 |
|  |  | Mean | 25.7 ± 7.7 | 39.5 ± 6.5 | <0.001 |
|  | 3-6 mm | Superior | 31.4 ± 11.3 | 45.4 ± 9.0 | <0.001 |
|  |  | Temporal | 19.8 ± 5.5 | 30.9 ± 7.9 | <0.001 |
|  |  | Inferior | 27.0 ±13.2 | 41.9 ± 7.5 | <0.001 |
|  |  | Nasal | 38.9 ± 13.9 | 54.6 ± 11.0 | <0.001 |
|  |  | Mean | 29.3 ± 10.3 | 43.2 ± 6.9 | <0.001 |
|  | 6-9 mm | Superior | 26.7 ± 10.3 | 38.5 ± 9.0 | <0.001 |
|  |  | Temporal | 15.5 ± 3.3 | 22.1 ± 8.2 | <0.001 |
|  |  | Inferior | 27.4 ± 11.8 | 38.4 ± 7.4 | <0.001 |
|  |  | Mean | 23.2 ± 7.8 | 33.0 ± 5.7 | <0.001 |
|  | 9-12 mm | Superior | 25.5 ± 8.0 | 25.3 ± 8.2 | 0.925 |
|  |  | Temporal | 13.3 ± 2.7 | 14.7 ± 2.5 | 0.048 |
|  |  | Inferior | 17.7 ± 6.9 | 19.1 ± 5.9 | 0.389 |
|  |  | Mean | 18.8 ± 5.1 | 19.7 ± 4.6 | 0.494 |
| DVC vessel density, mean ± SD, % | 1-3 mm | Superior | 54.4 ± 8.7 | 54.5 ± 5.3 | 0.953 |
|  |  | Temporal | 49.3 ± 12.4 | 53.2 ± 7.0 | 0.133 |
|  |  | Inferior | 46.6 ± 15.6 | 51.0 ± 7.4 | 0.170 |
|  |  | Nasal | 50.7 ± 11.0 | 53.3 ± 6.4 | 0.271 |
|  |  | Mean | 50.3 ± 10.2 | 53.0 ± 5.2 | 0.192 |
|  | 3-6 mm | Superior | 49.9 ± 8.6 | 46.8 ± 7.5 | 0.146 |
|  |  | Temporal | 53.4 ± 8.2 | 53.2 ± 6.9 | 0.897 |
|  |  | Inferior | 39.6 ± 16.3 | 41.8 ± 8.8 | 0.516 |
|  |  | Nasal | 49.3 ± 10.1 | 47.3 ± 9.2 | 0.410 |
|  |  | Mean | 48.1 ± 9.1 | 47.3 ± 4.8 | 0.672 |
|  | 6-9 mm | Superior | 40.2 ± 8.9 | 34.6 ± 8.4 | 0.015 |
|  |  | Temporal | 44.7 ± 9.9 | 42.9 ± 9.8 | 0.460 |
|  |  | Inferior | 27.6 ± 9.8 | 27.3 ± 10.5 | 0.926 |
|  |  | Mean | 37.5 ± 7.1 | 34.9 ± 6.2 | 0.139 |
|  | 9-12 mm | Superior | 30.7 ± 8.6 | 30.8 ± 7.9 | 0.969 |
|  |  | Temporal | 34.3 ± 11.2 | 32.1 ± 11.7 | 0.450 |
|  |  | Inferior | 23.6 ± 9.3 | 25.4 ± 8.3 | 0.416 |
|  |  | Mean | 29.5 ± 6.3 | 29.4 ± 6.8 | 0.953 |
| Foveal avascular zone area, mean ± SD, mm^2^ | | | 0.46 ± 0.15 | 0.28 ± 0.07 | <0.001 |

Abbreviation: PACG, primary angle-closure glaucoma; RNFL, retinal nerve fiber layer; GC-IPL, ganglion cell-inner plexiform layer; GCC, ganglion cell complex; SVC, superficial vascular complex; DVC, deep vascular complex; SD, standard deviation.

* Statistical differences were assessed by independent samples *t*-test.

**Table S2. Comparison of Peripapillary Optical Coherence Tomography Angiography Characteristics between PACG and Healthy Controls**

| Items | Regions | PACG  (n=30 eyes) | Healthy control  (n=30 eyes) | *P* Value* |
| --- | --- | --- | --- | --- |
| RNFL thickness,  mean ± SD, μm | Nasal superior | 87.1 ± 34.1 | 112.3 ± 27.9 | 0.003 |
|  | Nasal inferior | 75.1 ± 30.4 | 91.1 ± 24.5 | 0.029 |
|  | Inferior nasal | 118.9 ± 58.8 | 145.7 ± 36.6 | 0.038 |
|  | Inferior temporal | 123.9 ± 64.2 | 171.8 ± 44.3 | 0.001 |
|  | Temporal inferior | 73.8 ± 25.8 | 109.0 ± 15.5 | <0.001 |
|  | Temporal superior | 71.6 ± 21.1 | 105.9 ± 15.5 | <0.001 |
|  | Superior temporal | 107.0 ± 48.0 | 146.4 ± 36.7 | 0.001 |
|  | Superior nasal | 124.4 ± 60.7 | 151.4 ± 39.5 | 0.045 |
|  | Mean | 97.7 ± 39.7 | 129.2 ± 21.3 | <0.001 |
| GC-IPL thickness,  mean ± SD, μm | Nasal superior | 35.5 ± 4.7 | 36.0 ± 5.2 | 0.667 |
|  | Nasal inferior | 35.8 ± 4.1 | 37.4 ± 6.4 | 0.260 |
|  | Inferior nasal | 33.1 ± 4.7 | 32.2 ± 3.7 | 0.402 |
|  | Inferior temporal | 33.7 ± 4.5 | 32.8 ± 4.5 | 0.461 |
|  | Temporal inferior | 40.8 ± 6.6 | 38.6 ± 4.7 | 0.135 |
|  | Temporal superior | 41.4 ± 6.6 | 38.4 ± 5.1 | 0.059 |
|  | Superior temporal | 35.7 ± 3.8 | 34.3 ± 4.0 | 0.166 |
|  | Superior nasal | 33.9 ± 4.9 | 33.6 ± 4.2 | 0.765 |
|  | Mean | 36.2 ± 3.2 | 35.4 ± 2.9 | 0.301 |
| GCC thickness,  mean ± SD, μm | Nasal superior | 122.6 ± 34.2 | 148.3 ± 27.1 | 0.002 |
|  | Nasal inferior | 110.9 ± 30.0 | 128.5 ± 24.5 | 0.016 |
|  | Inferior nasal | 152.0 ± 58.8 | 177.9 ± 36.9 | 0.045 |
|  | Inferior temporal | 157.6 ± 64.0 | 204.6 ± 45.6 | 0.002 |
|  | Temporal inferior | 114.6 ± 27.4 | 147.6 ± 14.3 | <0.001 |
|  | Temporal superior | 113.0 ± 24.6 | 144.3± 16.5 | <0.001 |
|  | Superior temporal | 142.7 ± 49.6 | 180.6 ± 37.4 | 0.001 |
|  | Superior nasal | 158.3 ± 62.1 | 185.0 ± 40.0 | 0.052 |
|  | Mean | 133.9 ± 40.8 | 164.6 ± 21.7 | 0.001 |
| SVC vessel density,  mean ± SD, % | Nasal superior | 41.7 ± 11.5 | 52.3 ± 11.5 | 0.001 |
|  | Nasal inferior | 33.0 ± 12.2 | 41.7 ± 11.8 | 0.007 |
|  | Inferior nasal | 48.1 ± 16.0 | 57.9 ± 11.8 | 0.009 |
|  | Inferior temporal | 51.3 ± 19.2 | 69.7 ± 12.4 | <0.001 |
|  | Temporal inferior | 36.6 ± 13.8 | 53.8 ± 8.5 | <0.001 |
|  | Temporal superior | 35.2 ± 13.2 | 52.6 ± 11.0 | <0.001 |
|  | Superior temporal | 48.3 ± 17.9 | 62.8 ± 14.0 | 0.001 |
|  | Superior nasal | 51.6 ± 15.7 | 59.6 ± 11.6 | 0.030 |
|  | Mean | 43.2 ± 12.8 | 56.3 ± 6.8 | <0.001 |
| DVC vessel density, mean ± SD, % | Nasal superior | 26.3 ± 6.1 | 28.5 ± 8.9 | 0.285 |
|  | Nasal inferior | 28.9 ± 10.4 | 34.7 ± 10.3 | 0.036 |
|  | Inferior nasal | 15.8 ± 7.8 | 21.9 ± 8.0 | 0.004 |
|  | Inferior temporal | 18.5 ± 9.1 | 18.3 ± 10.3 | 0.935 |
|  | Temporal inferior | 39.2 ± 12.5 | 41.9 ± 11.2 | 0.374 |
|  | Temporal superior | 41.9 ± 13.3 | 43.2 ± 10.8 | 0.683 |
|  | Superior temporal | 25.2 ± 9.5 | 25.1 ± 12.1 | 0.961 |
|  | Superior nasal | 19.2 ± 9.4 | 21.4 ± 9.7 | 0.368 |
|  | Mean | 26.9 ± 5.7 | 29.4 ± 6.2 | 0.112 |

Abbreviation: PACG, primary angle-closure glaucoma; RNFL, retinal nerve fiber layer; GC-IPL, ganglion cell-inner plexiform layer; GCC, ganglion cell complex; SVC, superficial vascular complex; DVC, deep vascular complex; SD, standard deviation.

* Statistical differences were assessed by independent samples *t*-test.

**Table S3. Demographic and Clinical Characteristics of the Targeted Metabolomics Subset**

| Items | PACG  (n=20 eyes) | Healthy control  (n=20 eyes) | *P* Value |
| --- | --- | --- | --- |
| Age, mean ± SD, y | 56.6 ± 6.8 | 58.7 ± 8.6 | 0.397¶ |
| Male gender, n (%) | 3/20 (15.0%) | 6/20 (30.0%) | 0.451† |
| Hypertension, n (%) | 1/20 (5.0%) | 1/20 (5.0%) | >0.999† |
| Diabetes mellitus, n (%) | 1/20 (5.0%) | 2/20 (10.0%) | >0.999† |
| Body mass index, mean ± SD, kg/m^2^ | 23.2 ± 2.2 | 23.6 ± 1.5 | 0.547¶ |
| Intraocular pressure, mean ± SD, mmHg | 20.7 ± 2.7 | 14.0 ± 2.5 | <0.001¶ |
| Axial length, mean ± SD, mm | 22.8 ± 0.1 | 23.1 ± 0.2 | <0.001¶ |
| Anterior chamber depth, mean ± SD, mm | 1.8 ± 0.3 | 2.5 ± 0.3 | <0.001¶ |
| Lens thickness, mean ± SD, mm | 4.8 ± 0.2 | 4.3 ± 0.1 | <0.001¶ |
| Visual field mean deviation, mean ± SD, dB | -2.4 ± 1.4 | - | NA |

Abbreviation: PACG, primary angle-closure glaucoma; NA, not applicable; SD, standard deviation.

¶Statistical differences were assessed by independent samples *t*-test.

†Statistical differences were assessed by χ^2^ test or Fisher exact test, as appropriate.
